# Progesterone receptor membrane component 1 (PGRMC1) regulates Heme trafficking through mitochondria-ER junctions^☆^

**DOI:** 10.1016/j.jinorgbio.2025.113093

**Published:** 2025-10-07

**Authors:** Robert B. Piel, Chibuike D. Obi, Martonio Ponte Viana, Mathilda M. Willoughby, Osiris Martinez-Guzman, Aaliyah Wadley, Yasaman Jami-Alahmadi, James A. Wohlschlegel, Kevin G. Hicks, Jared Rutter, J. Alan Maschek, J. Leon Catrow, James Cox, Amit R. Reddi, Oleh Khalimonchuk, Amy E. Medlock

**Affiliations:** a Department of Biochemistry and Molecular Biology, University of Georgia, Athens, GA 30602, USA; b Department of Biochemistry, University of Nebraska, Lincoln, NE 68588, USA; c School of Chemistry and Biochemistry and School of Biological Sciences, Georgia Institute of Technology, Atlanta, GA 30332, USA; d Department of Biological Chemistry, University of California, Los Angeles, CA 90095, USA; e Department of Biochemistry University of Utah School of Medicine, Salt Lake City, UT 84112, USA; f Department of Nutrition and Integrative Physiology, University of Utah College of Health, Salt Lake City, UT 84112, USA; g Howard Hughes Medical Institute, University of Utah School of Medicine, Salt Lake City, UT 84112, USA; h Metabolomics Core Research Facility, University of Utah, Salt Lake City, UT 84112, USA; i Parker Petit Institute for Bioengineering and Biosciences, Georgia Institute of Technology, Atlanta, GA 30332, USA; j Nebraska Redox Biology Center, University of Nebraska, Lincoln, NE 68588, USA; k Fred & Pamela Buffett Cancer Center, Omaha, NE 68198, USA; l Department of Interdisciplinary Biomedical Sciences, University of Georgia School of Medicine, Athens, GA 30602, USA

**Keywords:** Mitochondria, Heme, Ferrochelatase, PGRMC1, MAMs

## Abstract

Heme is a cofactor essential for a multitude of biological reactions. The terminal step of heme synthesis occurs in the mitochondrial matrix which means that heme must be trafficked from there to other locales in the cell. Thus, identifying intracellular heme chaperones is crucial to understanding regulation of global cellular metabolism. The heme-binding protein progesterone receptor membrane component 1 (PGRMC1) has been proposed to function as a chaperone for several biologically active molecules including heme, but its cellular role is not fully understood. Here, we investigate the function of PGRMC1 in heme metabolism. By monitoring intracellular heme location and concentrations in *Saccharomyces cerevisiae,* we show that mutants lacking damage associated protein 1 (Dap1), the yeast ortholog of PGRMC1, have altered nuclear heme trafficking which can be corrected by complementation with *DAP1* or *PGRMC1*. Biochemical analyses reveal that PGRMC1 co-localizes with known mitochondrial-associated membrane (MAM) proteins and proteomic comparison of interaction partners shows enrichment of MAM-associated proteins and pathways. Metabolomics profiling of wild-type and PGRMC1 knockout cells identifies significant changes of several metabolites, including heme, several amino acids, long chain acyl-carnitine, ethanolamine phosphate, and mevalonic acid. Together, these results provide evidence that PGRMC1 is involved in heme trafficking and homeostasis through MAMs.

## 1. Introduction

Heme is an essential cofactor in eukaryotes, participating in a variety of cellular processes. While the pathway for heme synthesis in eukaryotes is well defined [1], the mechanisms by which heme is moved from its site of synthesis, the mitochondrial matrix, to other cellular locales are unclear. What is known is that heme is a potentially cytotoxic molecule that does not exist within cells as a free molecule in significant concentrations [2-4]. This suggests the presence of a systematic trafficking system or systems to protect the cellular milieu. One of several proteins proposed to play a role in regulating heme synthesis and heme trafficking is progesterone receptor membrane component 1 (PGRMC1) [5-7].

PGRMC1 belongs to the membrane-associated progesterone receptor (MAPR) family. MAPR proteins are distant homologs of cytochrome *b*5, and all share a cytochrome *b*5-like domain. In addition to PGRMC1, other MAPR proteins in mammalian cells include progesterone receptor membrane protein 2 (PGRMC2), Neudesin, and Neuferricin. PGRMC2 is most similar to PGRMC1 in that it has a proposed amino terminal transmembrane domain, while Neudesin and Neuferricin are soluble, secreted proteins [8]. PGRMC2 has been shown to interact with PGRMC1 [5,7] and to function in heme homeostasis [5]. PGRMC1 has been called an ‘enigmatic heme-binding protein’ since its reported cellular roles are broad, including heme homeostasis, regulation of cytochrome P450s, iron homeostasis, reticulopathy, regulation of calcium storage, cell surface signaling, progesterone signaling, and lipid and glucose metabolism [9].

Along with the poorly defined cellular functions of PGRMC1, questions exist related to basic properties of the protein such as ligand binding and cellular localization. While the ability of PGRMC1 to bind heme is well established, the exact mechanism of heme binding and release are currently unclear. Studies on PGRMC1 and its yeast ortholog, damage associated protein 1 (Dap1), have shown that both preferentially bind ferric heme [6] with reported affinities for heme binding by PGRMC1 ranging from nM [10] to μM [11] and for Dap1 pM [12]. Heme binding by PGRMC1 is by conserved active site residues, specifically tyrosine 113, lysine 163 and tyrosine 164 (human numbering) [10,11]. It is of note that the PGRMC1 protein employed for affinity measurements was a truncated protein that lacked the amino terminal putative transmembrane domain. In contrast full length Dap1 was used for most biophysical studies, but of note is that Dap1 lacks a putative transmembrane domain. Likewise, crystal structures determined for heme-bound PGRMC1 were obtained with the truncated, soluble portion of the protein [10]. Given that all biophysical studies of PGRMC1 were done with the truncated, soluble form of the protein, the role that the amino terminus and predicted transmembrane domain may play in protein oligomerization and heme binding and release remains unclear.

In vivo and in vitro studies focused on identifying the nature of interactions involving PGRMC1 have provided some clues to the source of heme for PGRMC1. We and others determined that PGRMC1 is a protein partner of the terminal enzyme of the heme biosynthetic pathway, ferrochelatase (FECH) [7,11], with preferential interaction to the heme bound form of FECH, and regulates FECH activity in vitro [7]. PGRMC1 has a moderate affinity for heme and is able to participate in vitro in heme transfer either to or from other hemoproteins. These findings along with those for PGRMC2 [5] are consistent with PGRMC1 playing a role in cellular heme homeostasis. Given the diversity of cellular processes [13] or roles as a regulatory molecule [14,15] for which heme is required, the role of PGRMC1 in heme synthesis and trafficking could provide a link to many of the ascribed functions of PGRMC1.

The identified interactions between PGRMC1 and FECH highlight another area of uncertainty, the subcellular localization of PGRMC1. Existing reports provide conflicting information regarding the subcellular localization of PGRMC1 [16]. While some studies suggest that PGRMC1 resides in the mitochondrial outer membrane (OM) [7], others have proposed it to be localized to the ER [17]. Recent high-throughput proximity labeling studies [18-20] indicate a more likely scenario, wherein PGRMC1 localizes at the interface of these two organelles, specifically mitochondrial-associated membranes (MAMs). MAMs are known to mediate ion, lipid, and metabolite exchange between the ER and mitochondrial networks [21-23] and given that several of these functions have been ascribed to PGRMC1, subcellular localization at these locales may help to explain the many recognized roles of PGRMC1 in normal and disease states [9]. To better understand the role played by PGRMC1 in metabolic and pathophysiological processes, we have defined the subcellular localization and interactome of PGRMC1, as well as examined the metabolic consequences of PGRMC1 deficiency. Additionally, we investigated the role of Dap1 and PGRMC1 in cellular heme trafficking in yeast cells and identified metabolites that bind to PGRMC1. Taken together, our results support a key role for PGRMC1 at MAMs and in heme trafficking and homeostasis.

## 2. Experimental

### 2.1. S. cerevisiae genetic rescue experiments and heme trafficking assay

*S. cerevisiae* strains used in this study were derived from the BY4741 (*MAT*a, *his3*Δ1, *leu2*Δ0, *met15*Δ0, *ura3*Δ0) genetic background. The *dap1*∷*KANMX4* deletion strain was obtained from the yeast knockout library *(Horizon Discovery)* and confirmed by sequencing the unique barcodes flanking the KanMX4 deletion cassette and validating its fluconazole sensitivity [12]. The pRS413-GPD plasmid [24] was used for expression of full-length N-terminal HA-tagged *DAP1* and human *PGRMC1* in complementation experiments. A 5′ Xba1 and 3’ *Spe*I fragment of HA-Dap1 or HA-PGRMC1 was amplified from yeast genomic DNA or from the plasmid PGRMC1 pTrc1His [7], respectively, using the Dap1 or PGRMC1 primers indicated below and ligated into pRS413-GPD.

HA-Dap1_Forward Primer.
5’AGTCATATGTCTAGAATGTACCCATACGATGTTCCA-GATTACGCTTCCTTCATTAAAAACTTGTTATTTGGAGGTGTT3’
HA-Dap1_Reverse Primer.
5’ATAAAAATTACTAGTTCATACGTTCACGCCAGGCTCCGGAATCA-GAGTA3’
HA-PGRMC1_Forward Primer.
5’CATGGTATGTCTAGAATGTACCCATACGATGTTCCA-GATTACGCTGCTGCCGAGGATGTGGTGGCGACT3’
HA-PGRMC1_Reverse Primer.
5’AAAA-CAGCCACTAGTTTAATCATTTTTCCGGGCACTCT-CATCTTTTGGTTCTTCCTCATCTGAGTA3’

Previously described pRS415-GPD plasmids expressing GPD-driven cytosolic, mitochondrial, and nuclear localized heme sensors were introduced into the indicated WT and *dap1*Δ strains [2,25,26]. Yeast transformations were performed using the lithium acetate procedure [27]. Strains were maintained at 30 °C on either enriched yeast extract (1 %) and peptone (2 %) based medium supplemented with 2 % glucose (YPD), or synthetic complete medium (SC; Sunrise Science Products) supplemented with 2 % glucose and the appropriate amino acids to maintain selection. Cells cultured on solid medium plates were done so with YPD or SC media supplemented with 2 % agar [2]. Selection for yeast strains containing the *KanMX4* marker was done on YPD agar plates supplemented with G418 (200 μg/mL) [2]. To assess fluconazole sensitivity, WT and *dap1*Δ strains were cultured with and without 10 μg/mL Fluconazole (Sigma). Steady state levels of labile heme, total heme, and heme trafficking dynamics assays were carried out exactly as previously described [25,26,28].

### 2.2. Affinity purification cell lines, growth conditions, sample preparation and analysis

Human PGRMC1 FLAG-tagged construct was designed as previously reported [7]. Three mammalian cell lines were used for PGRMC1 interaction studies: murine erythroleukemia (MEL) cells [29,30] (a kind gift of the late Barry Paw, Harvard University), human embryonic kidney 293 T (HEK293T) cells (ATCC, CRL-3216), and human hepatocellular carcinoma (HepG2) cells (ATCC, HB-8065). Cells were transfected with either a FLAG-tagged PGRMC1 expression vector or empty vector by electroporation and stably expressing cell lines were selected for puromycin resistance (5 μg/mL) as previously described [31]. For HepG2 cells, their post-transfection recovery time was 3–4 weeks, as compared to 1–2 weeks for MEL and HEK293T cells. Cells stably expressing FLAG-tagged PGRMC1 were validated by immunoblot analysis using anti-FLAG antibody (F7425, Sigma). MEL and HEK293T cells were cultured in DMEM with 25 mM glucose, 1 mM sodium pyruvate and 4 mM glutamine (Cellgro), supplemented with 10 % FBS (Atlanta Biologicals) and 1 % penicillin/streptomycin (Cellgro). HepG2 cells were cultured in RPMI 1640 with l-glutamine (Cellgro), supplemented with sodium pyruvate, 10 % FBS (Atlanta Biologicals) and 1 % penicillin/streptomycin (Cellgro). Cells stably expressing constructs were grown in media containing 5 μg/mL puromycin to about 95 % confluency (1.5 × 10^6^ cells in ten 175cm^2^ flasks), harvested, and lysed for mitochondria isolation [32]. Isolated mitochondria were lysed for affinity purification of FLAG-tagged PGRMC1 as previously described [31]. Biological replicates from all cell lines were done in duplicate.

Protein preparation, mass spectrometry, and data analysis were carried out as previously described [33], including the use of the Contaminant Repository for Affinity Purification (CRAPome) [34] to analyze the enrichment of PGRMC1 interacting partners (prey) in our study cell lines (supplemental Table S1). The enrichment profiles of PGRMC1 prey in different cell lines were represented as maximum fold change, herein referred to as fold change (FC). Uniprot gene names are used throughout.

For all subsequent analysis of prey, a FC threshold was placed for the selection of highly enriched proteins in each dataset. These threshold values were 2 for HEK293T cells, and 1.5 for MEL and HepG2 cells. Overall HepG2 and MEL enrichment profile was lower than the other cell line, thus, leading to the variation in selected threshold values. JVenn was used to create a Venn diagram of prey from different cell lines [35]. Subcellular localization of PGRMC1 prey was classified using SubCell BarCode [36], MitoCarta 3.0 [37], and g:Profiler [38]. To analyze the biological pathway enrichment of prey proteins g:Profiler [38] was used. Biological processes are indicated in the text in italics.

Additionally, since calculated FC values of PGRMC1 prey were different in the different cell types, we normalized the FC value of prey proteins for comparison of bait-prey interactions between cell lines. Specifically, the highest FC values in MEL, HEK293T, and HepG2 cells were 55, 30, and 9, respectively. Normalized FC per cell line were calculated as shown below.


Normalized cell A protein FC=log((Protein FC for cell A∕Highest cell A FC)x100)

This normalized FC were used to generate cell line-dependent visualization of PGRMC1-prey interactions using DISPLYR (Pyrmont) to plot heatmaps.

### 2.3. Subcellular fractionations

Subcellular fractionations were carried out largely as described in Wieckowski et al. [39] with some modifications. Briefly, the livers isolated from two 24 h-fasted C57BL/6 mice (a kind gift of Dr. Edward Harris, UNL) were immediately transferred to cold IB1 buffer (225 mM mannitol, 75 mM sucrose, 0.5 % BSA, 0.5 mM EGTA and 30 mM Tris-HCl, pH 7.4). The livers were diced in cold IB3 buffer (225 mM mannitol, 75 mM sucrose and 30 mM Tris-HCl, pH 7.4) and later washed in the same buffer to remove blood. The liver pieces were then washed again in cold IB1 buffer. The washed liver fragments were transferred to potter glassware and 4 mL of IB1 buffer per gram of liver tissue were added. The tissue was homogenized using motorized Teflon pestle by 8 strokes at 1500 rpm. The homogenate was centrifuged at 740 x*g* for 5 min at 4 °C. The supernatant was recovered and centrifuged again. Aliquots of the nuclei-enriched pellets were kept for further analysis. Cleared supernatant was then centrifuged at 9000 x*g* for 10 min at 4 °C. The supernatant fractions rich in cytoplasmic proteins and lysosomes were saved for subsequent analysis. The pellets were soaked in 20 mL of IB2 buffer (225 mM mannitol, 75 mM sucrose, 0.5 % BSA, and 30 mM Tris-HCl, pH 7.4) and subjected to centrifugation at 10,000 x*g* for 10 min at 4 °C. This step was repeated twice. The crude mitochondrial pellets were solubilized in 2 mL of mitochondria-resuspending buffer (MRB, 250 mM mannitol, 5 mM HEPES, pH 7.4, and 0.5 mM EGTA). Eight milliliters of Percoll buffer (225 mM mannitol, 25 mM HEPES, pH 7.4, 1 mM EGTA, and 30 % *v*/v Percoll) were placed inside of a 14 mL polyallomer tube, and covered with 2 mL of the mitochondria suspension. Additional 3.5 mL of MRB were added atop of the mitochondria suspension and the samples were fractionated at 95,000 x*g* for 30 min at 4 °C. Two visible ring-shaped fractions were recovered: a lower one harboring purified mitochondria, and the top one enriched in MAMs. These fractions were collected in different tubes, diluted 10-fold with MRB, and centrifuged at 6300 x*g* for 10 min at 4 °C. The MAM-enriched supernatants were subjected to yet another centrifugation step at 100,000 x*g* for 1 h at 4 °C. Following centrifugation, the MAM pellets were resuspended in 200 μL of MRB. The supernatant from the mitochondrial fraction was discarded and the pellets were resuspended in 20 mL of MRB, centrifuged again at 6300 x*g* for 10 min at 4 ° C, and solubilized in 500 μL of MRB. Isolated fractions of interest were analyzed by immunoblotting. The following primary antibodies were used: rabbit anti-PGRMC1 (HPA002877, Sigma); mouse anti-IP3R (200–301-F77, Rockland Immunochemicals); rabbit anti-ATF6 (bs-1634R, Bioss Antibodies); rabbit anti-FACL4 (22401–1-AP, Proteintech), mouse anti-TOMM20 (sc-17,764, Santa Cruz Biotechnology); and rabbit anti-HSPD1 (15282–1-AP, Proteintech). Antibodies were tested for reliability to assure specificity of detection at expected migration distances.

### 2.4. MIDAS analysis

PGRMC1 protein for MIDAS analysis was produced as previously described [7] at a concentration of 200 μM. Samples were buffer exchanged with 150 mM Ammonium acetate, pH 7.4 prior to MIDAS analysis, which was carried out as previously described [40].

### 2.5. Crispr-Cas9 gene perturbation

Human erythroid tissue culture experiments were conducted in K562 human myelogenous leukemia (K562) cells (ATCC – CCL243) [41,42]. Cells were cultured in DMEM with 25 mM glucose, 1 mM sodium pyruvate and 4 mM glutamine (Cellgro) plus 10 % FBS (Atlanta Biologicals) and 1 % penicillin/streptomycin (Cellgro). For induction of K562 cells 1 mM sodium butyrate was included in growth media and cells were grown 6 days [43,44].

CRISPR-Cas9 mutagenesis in K562 cells utilized PX458 (Addgene, Plasmid #48138), a vector expressing all parts of CRISPR-Cas9 machinery and a GFP reporter gene [45]. A pair of oligonucleotides (Sigma) corresponding to each gRNA target site (supplemental Fig. S1a) and its reverse complement were annealed and cloned into PX458 using *Bbs*I (NEB) [45]. A single completed plasmid was transfected into K562 cells for each desired target site, with two plasmids being transfected simultaneously for generation of excision mutants. Transfection of plasmids into K562 cells was accomplished using X-tremeGENE HP DNA Transfection Reagent (Sigma) according to manufacturer’s instructions at a ratio of 1 μg plasmid DNA to 1 μL reagent. Single cells expressing PX458 constructs were identified and isolated at the University of Georgia Center for Tropical and Emerging Global Diseases Cytometry Shared Resource Core using the MoFlo XDP (Beckman Coulter) cell sorter screening for GFP fluorescence. To perform rescue experiments of PGRMC1 KO cells, PGRMC1 was expressed from the pEF1alpha FLAG biotag vector as previously described [7]. Single cells expressing the PGRMC1 rescue vector were selected via serial dilution and plating in media containing 5 μg/mL of puromycin (Cellgro).

Successful excision mutation of human PGRMC1 in K562 cells was verified using PCR (supplemental Fig. 1b). Genetic knockout (KO) was further verified via Sanger sequencing (Eton Bioscience Inc). Ablation of PGRMC1 protein expression in KO cells (supplemental Fig. S1c) relative to total protein and expression of PGRMC1 from the rescue vector was verified via immunoblot of cell lysate as previously described [7].

### 2.6. Porphyrin and heme analysis

Hemoglobin (Hb) content of intact K562 cells was determined using an Olis CLARiTY Spectrophotometer (Olis) as previously described [46]. Cell counts were taken using Scepter handheld automated cell counter (Millipore) using the 60 μm tip. Heme, protoporphyrin IX, Zinc-protoporphyrin IX, and total porphyrin levels in K562 cells were measured via ultra-performance liquid chromatography as previously described [31,47]. Results are presented as mean ± SD. Unpaired *t-*test was carried out using GraphPad (https://www.graphpad.com/quickcalcs/ttest1/) for statistical analysis.

### 2.7. Metabolomics analyses

Liquid chromatography-mass spectrometry (LC-MS) and gas chromatography with flame ionization detection (GC-FID) were implemented for metabolomic analysis of intracellular compounds from K562 wild-type (*n* = 4) and PGRMC1 null cells (*n* = 3 for LC-MS and n = 4 for GC-FID). From 100 μL samples of ~15 million cells, 75 μL was removed for LC-MS metabolomics. 75 μL of cells was extracted with 300 μL of ice cold 75 % ACN in H_2_O + 0.1 % ammonium hydroxide containing 0.1 μg/mL d9-carnitine and 1 μg/mL of d4-tyrosine internal standards. A process blank was created at this time containing the extraction solvent and 75 μL of H_2_O. Samples were vortexed for 30 s then sonicated on ice for 2 min. Samples were chilled at −20 °C for 1 h and then centrifuged at 20,000 x*g* for 10 min at 4 °C. The supernatant was collected and transferred to new Eppendorf tubes. To remove lipids, 750 μL of cold MTBE and 50 μL of cold H_2_O + 0.1 % ammonium hydroxide was added to each sample. Samples were vortexed, then frozen at −80 °C for 30 min. The MTBE layer was discarded and the aqueous layer dried overnight under vacuum. Samples were reconstituted with 300 μL 50 % ACN in H_2_O prior to analysis.

Prior to analysis, samples and process blanks were transferred to PTFE autosampler vials. A pooled quality control (QC) sample was prepared by combining 5 μL of each sample. Samples were tested in replicates, and the order was randomized prior to analysis. An Agilent 6545 UPLC-QToF (Agilent Technologies) run in both positive and negative modes was used for analysis. Separation was achieved using a Sequant Zic-pHILIC 2.1 × 100 mm column (Millipore Sigma) with Phenomenex Krudkatcher (Phenomenex). An initial concentration of 99 % ACN with 5 % ddH_2_O and 5 % 25 mM ammonium carbonate in ddH2O was held for 1 min at a flow rate of 0.2. B was decreased to 10 % over 14 min and held for 5 min. B was returned to starting conditions over 0.1 min, and the system was allowed to re-equilibrate for 10 min between runs at a flow rate of 0.2 mL/min.

Data was collected using MassHunter software (Agilent). Molecular features were identified using MassHunter Profinder 10.0 and their peak area was recorded using MassHunter Quant 9.0. This data was transferred to an Excel spread sheet (Microsoft). Metabolite identity was established using a combination of an in-house metabolite library developed using pure purchased standards and the METLIN library (https://metlin.scripps.edu/landing_page.php?pgcontent=mainPage). Data and statistical analysis were performed using MetaboAnalystR [48].

The remaining 25 μL of cell, used for GC-FID, was transferred to 13 × 100 mm glass tubes with PTFA lined cap containing 1 mL of 5 % HCl in MeOH with internal standards FA 13:0 at 5 μg/mL, FA 15:0 at 50 μg/mL, FA 17:0 at 150 μg/mL and FA 23:0 at 80 μg/mL. Samples were briefly vortexed then placed into a sand bath heated at 80 °C for 2 h. The resulting methanolysis reaction products were extracted with hexanes (2 × 1 mL), transferred to 13 × 100 mm glass tubes, briefly evaporated in a speedvac, resuspended in 250 μL EtOAc and transferred to GC/MS vials with insert. A pooled QC sample was generated by taking small aliquots from each reaction vial. A blank extraction process sample was also prepared. GC-FID analyses are conducted using an HP6890 instrument interfaced with a flame ionization detector and equipped with a DB-23 (60 m × 0.25 mm ID, 0.15 μm film thickness; Agilent Technologies, Inc.) column and an HP7682 injector. Helium is used as a carrier gas with a 10:1 split ratio at an injection volume of 1 μL. The injector temperature is 250 °C. The oven temperature gradient was programmed as follows: 50 °C held for 1 min, increased at a rate of 25 °C/min to 175 °C, increased at a rate of 4 °C/min to 230 °C and held for 10 min. Detector temperature set at 280 °C. Hydrogen: 40 mL/min; air: 450 mL/min; helium make-up gas: 30 mL/min. Samples were normalized to FA 13:0 based on peak ratio to the IS. Data and statistical analysis were performed using MetaboAnalystR [48].

## 3. Results and discussion

### 3.1. Role of PGRMC1 in heme trafficking in S. cerevisiae

**Fig. 1.**
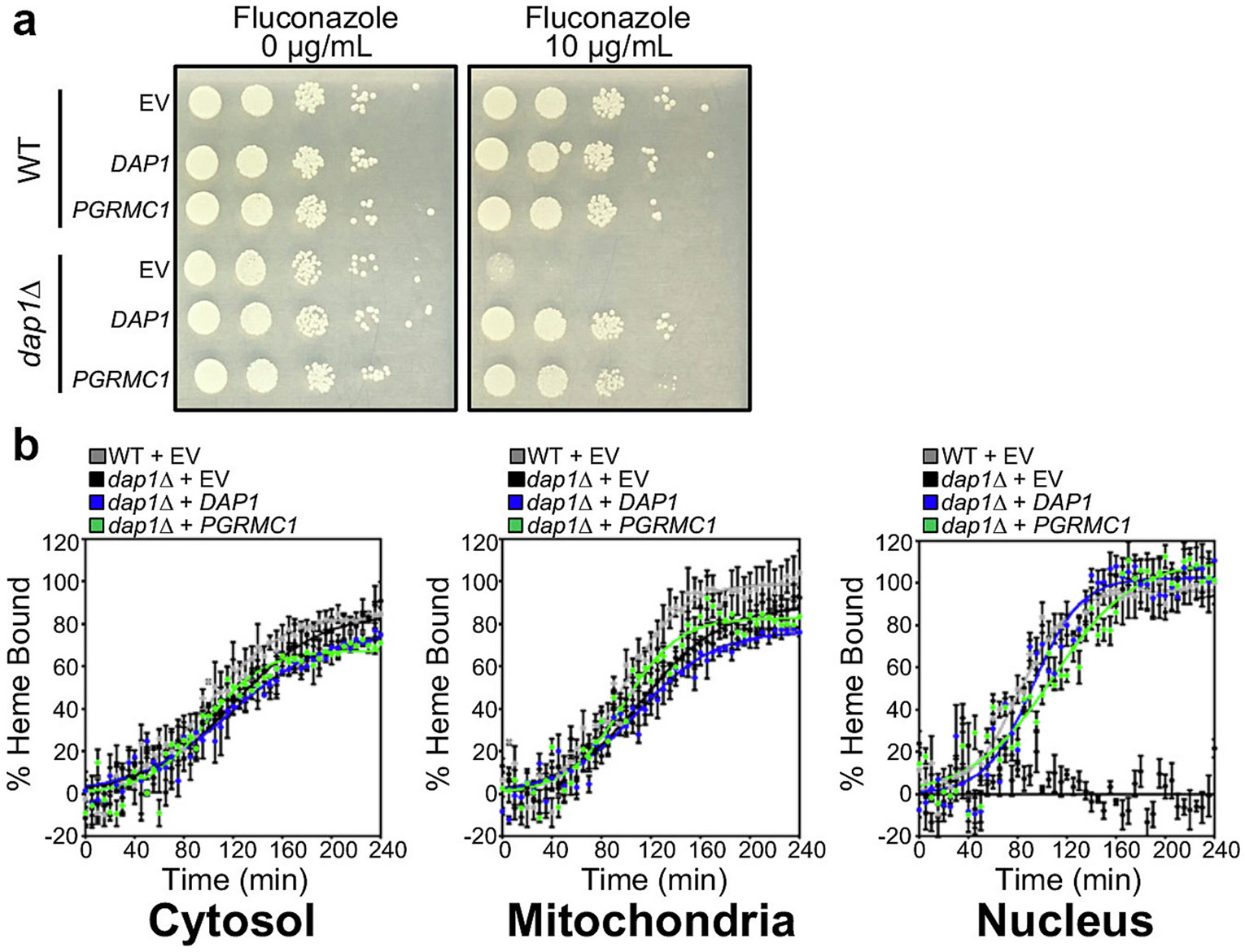
Complementation of PGRMC1 in *dap1*Δ cells and effect on intracellular heme trafficking. (a) Fluconazole susceptibility growth test of WT and *dap1*Δ cells expressing vector control (EV), *S. cerevisiae DAP1* (*DAP1*), or human *PGRMC1* (*PGRMC1*). Cells were spotted in YPD media without fluconazole (0 μg/mL) or with (10 μg/mL) and cultured for 3 days at 30 ° C. (b) WT expressing HS1 and vector control (EV) (grey) as well as *dap1*Δ cells expressing HS1 plus vector control (EV) (black), *DAP1* (blue), or *PGRMC1* (green) were depleted of heme by treatment succinyl acetone. Upon removal of succinyl acetone and re-initiation of heme synthesis the rates of heme trafficking to the cytosol (left), the mitochondrial matrix (center), and nucleus (right) were measured as previously described [26].

We utilized the model organism *S. cerevisiae* to define the role PGRMC1 plays in heme homeostasis and trafficking. A robust system for measuring heme homeostasis and trafficking exists in *S. cerevisiae* [2,26] and Dap1, the *S. cerevisiae* ortholog of PGRMC1, has been characterized with respect to heme binding and its interactions and regulation of cytochrome P450s [6,49]. Since Dap1 lacks the predicted transmembrane domain found in PGRMC1, we first tested the ability of PGRMC1 to rescue *dap1*Δ cells in the presence of fluconazole, an Erg11 sterol demethylase inhibitor [50]. Complementation studies showed that both human PGRMC1 and yeast Dap1 rescue growth of *dap1*Δ cells in the presence of fluconazole (Fig. 1a), supporting functional equivalency of the two proteins in *S. cerevisiae*. Significantly, the steady state levels of labile heme in the cytosol as measured using the HS1 heme sensor [2,26] and total cellular heme content as measured using the oxalic acid method [25] did not differ between wild-type and *dap1*Δ cells (supplemental Fig. S2). While this was unanticipated based on the findings that PGRMC1 decreases FECH activity in vitro [7], it may be explained by differences in regulation of heme synthesis which are not well understood [51,52] or multiple means of heme export from the mitochondria [26]. Interestingly, in *dap1*Δ cells the rate of heme trafficking to the nucleus was found to be impaired, but there was not a corresponding change in heme trafficking rate to the cytoplasm or mitochondrial matrix. This decrease in nuclear heme trafficking was rescued by expression of *DAP1* or *PGRMC1* (Fig. 1b). Of note is that heme trafficking to the nucleus occurs at a rate 25 % faster than trafficking to other compartments [26]. Additionally, when ER-mitochondrial encounter structures, the equivalent of MAMs in yeast, are stabilized there is increased heme trafficking to the nucleus [26], thus further supporting a role of PGRMC1, likely associated with MAMs, in cellular heme homeostasis.

### 3.2. PGRMC1 interactome

The possibility that PGRMC1 is functioning in a MAM environment in mammalian cells was explored by determining if it was spatially associated with MAM-specific proteins. Our approach was to use affinity purification and mass spectrometry (AP-MS), an approach that has previously been employed in a differentiating erythroid cell line cells to identify PGRMC1’s interaction with FECH [7]. While this approach has limitation, it was an extension of our previous work and new tools exist to help filter datasets. Herein FLAG-PGRMC1 was the bait in three different cell lines to explore how protein interactions could differ based on cellular proteome and metabolism. Cell lines were HepG2 and HEK293T, human liver and kidney-derived cell lines, respectively, and MEL, a murine erythroid cell line. Cell lines were selected as they exhibit a range of heme synthesis levels, with heme synthesis being highest in MEL, less in HepG2 and lowest in HEK293T [53]. Since AP-MS experiments identified a large number of proteins from each cell line, the CRAPome database and analysis tool [34] was used to filter out common background contaminants including proteins which non-specifically bind to immunoglobulin molecules and solid-phase support in AP-MS experiments. Most interactions identified are not present in the BioGRID [54] or IntACT [55] databases. HEK293T cells had the highest number of enriched PGRMC1 prey proteins, 2930, while HepG2 had 1024 and MEL 644 prey proteins. To increase the stringency, a threshold on the FC values was placed on each cell line, specifically 1.5 for MEL and HepG2, and 2.0 for HEK293T cells. Thresholds were selected for each cell line based on FC calculations and the Significance Analysis of INTeractome (SAINT) score from CRAPome analysis [34]. Filtering resulted in 932 proteins in HEK293T cells, 336 in HepG2 cells, and 404 in MEL cells (supplemental Table S2) In addition, for a comparison between cell lines, FC values were normalized.

**Fig. 2.**
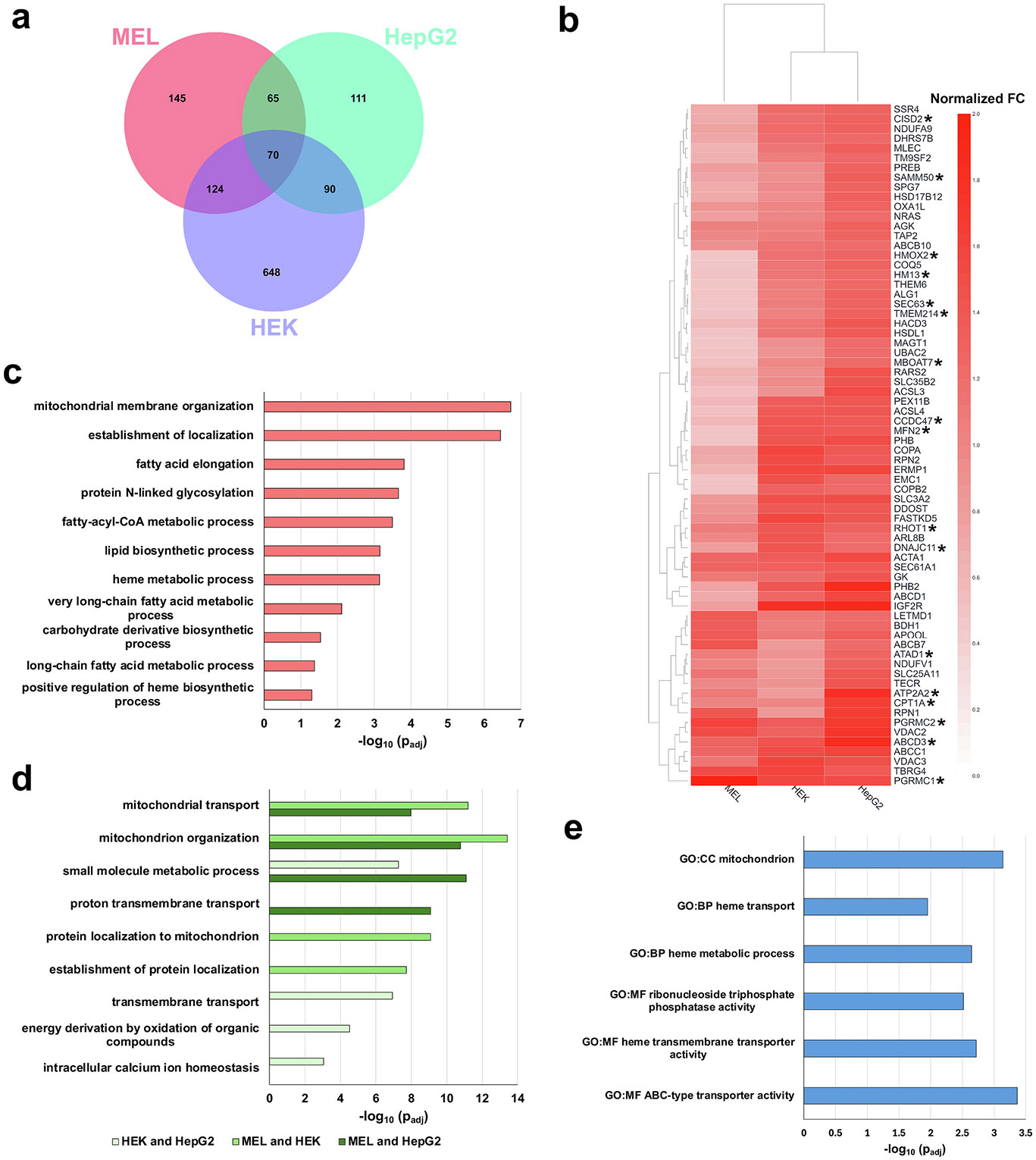
Comparative analysis of PGRMC1 interactome. (a) Venn diagram of identified PGRMC1 prey from MEL, HEK293T (HEK), and HepG2 cell lines filtered using the CRAPome repository. (b) Heatmap of PGRMC1 interactome common in all three cells lines using the normalized FC values, represented by color scale (2.0 to 0.0) with all values greater than 0.0. Prey proteins previously identified as candidate proteins of MAMs indicated with asterisk (*). (c) Bar graph of biological process enrichment profile of 70 proteins found in the core PGRMC1 interactome in MEL, HEK293T (HEK), and HepG2 cells. X-axis shows −log_10_ of adjusted *p*-value (p_adj_) and Y-axis the biological process. (d) Bar graph of pathway enrichment profile of proteins found in at least two cell lines. Top four biological processes shown for each cell line combination. X-axis shows −log_10_ of adjusted p-value and Y-axis the biological process. (e) Bar graph of gene ontology (GO), cellular compartment (CC), biological process (BP), and molecular function (MF) of common PGRMC1 and FECH interactome proteins. X-axis shows −log_10_ of adjusted p-value and Y-axis is GO aspect.

To define the core interactome of PGRMC1, prey common to all three cells lines were investigated in terms of subcellular location and biological pathway enrichment. A total of 70 proteins (4.2 % of the total proteins identified) were found in all three cell lines (Fig. 2a and supplemental Table S2). Normalized fold changes for these 70 proteins in each cell line are shown in the heatmap in Fig. 2b. Ten (14 %) of the 70 core interactome proteins have previously been shown to interact with PGRMC1 in either an erythroid cell line [7] or mouse liver [11], specifically ABCB7, ABCB10, ABCD3, ACSL1, APOOOL, ATP2A2, DDOST, SEC61A1, SLC25A11, and VDAC2. Investigation of the subcellular localization of core interactome proteins showed approximately ~39 % with sole localization to the mitochondria, ~40 % with sole localization to the ER, and ~ 9 % with localization reported for both the mitochondria and the ER. The localization of the remaining proteins was either reported as secretory pathway or is undefined (supplemental Table S2). Interestingly, 17 of the 70 prey proteins, ABCD3, ATAD1, ATP2A2, CCDC47, CISD2, CPT1A, DNAJC11, HM13, HMOX2, MBOAT7, MFRN2, PGRMC1, PGRMC2, RHOT1, SAMM50, SEC63, and TMEM214, have been identified as candidate proteins of MAMs (Fig. 2b) [18-20,56].

To explore the biological functions of the core PGRMC1 interactome proteins, pathway enrichment analysis was investigated using g:Profiler [38]. Biological processes which showed enrichment included *mitochondrial membrane organization*, *lipid biosynthetic processes*, and *heme metabolic processes* (Fig. 2c and supplemental Table S3). One protein of particular interest in heme metabolism is heme oxygenase 2 (HMOX2), the constitutively expressed heme degradation and heme binding protein [57], proposed to have a role in regulating the bioavailability of heme [58]. To expand on the interactome and further understand the biological processes in which PGRMC1 prey are involved we investigated pathway enrichment for proteins found in at least two cell lines (supplemental Table S2). The top four biological processes enriched for each combination of cell lines are shown in Fig. 2d. Pathway enrichment analyses support not only the core interactome with respect to *mitochondrion organization*, but also indicate additional roles including *transmembrane transport* and *intracellular calcium ion homeostasis*. Many of the biological processes enriched in the core and expanded interactome of PGRMC1 have been ascribed to putative functions of PGRMC1 [9]. Furthermore, and related to the subcellular localization, some of the indicated biological processes, specifically phospholipid synthesis and cellular calcium homeostasis, are known to occur at MAMs [23].

Some proteins previously shown to interact with PGRMC1 [7,11], namely FECH and some cytochrome P450s, were not enriched in multiple cell lines. This is likely attributable to low expression levels of these proteins. For FECH and other heme synthesis enzymes expression and protein levels increase when heme demand increases such as during erythropoiesis [53]. While not part of the core interactome, FECH was identified as a prey protein unique to the erythroid cell line as were some cytochrome P450s which were unique to the liver cell line (supplemental Table S2). With the recent identification of the core interactome of FECH [33], this allowed the comparison of the PGRMC1 and FECH core interactomes. A total of eleven proteins were found to be common between the interactomes, specifically ABCB7, ABCC1, ABCD3, ARL8B, ATAD1, BDH1, PGRMC1, PGRMC2, RHOT1, RPN1, and TBRG4 (supplemental Table S2). g:Profiler [38] analysis of this diverse group of proteins shows that most are localized to the mitochondria with biological process enrichment in heme metabolism and transport (Fig. 2e and supplemental Table S3). Overall, the finding from the PGRMC1 interactome analysis support its link to heme homeostasis and MAM-associated biological processes.

### 3.3. Subcellular localization of PGRMC1

**Fig. 3.**
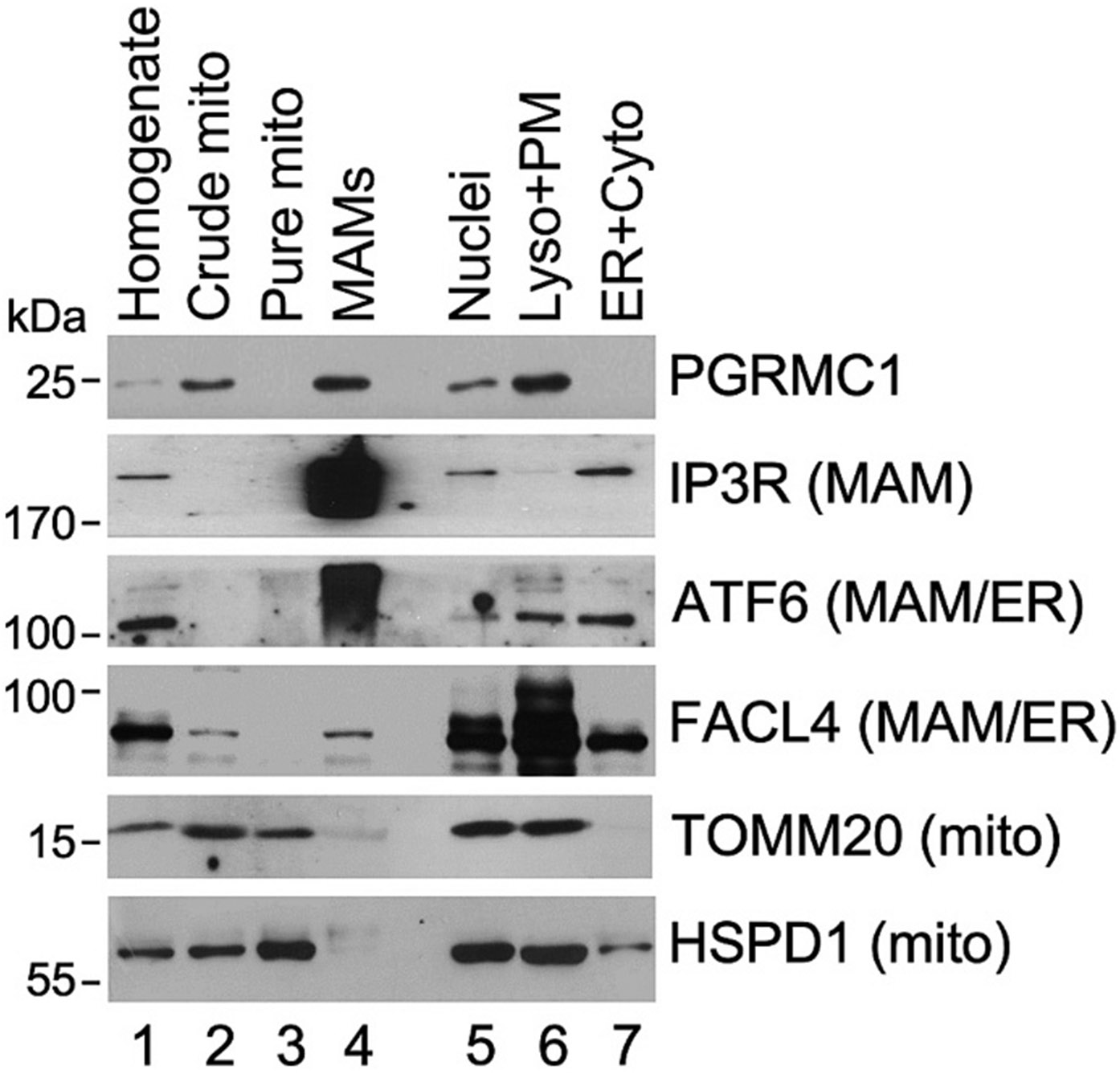
Subcellular localization of PGRMC1 with MAMs. Immunoblot of fractionated mouse livers. Fractions are liver homogenate (Homogenate), crude mitochondria (Crude mito), purified mitochondria (Pure mito), mitochondrial-associated membranes (MAMs), nuclear (Nuclei), lysosome and plasma membrane (Lyso + PM), and ER and cytosol (ER + Cyto). Fraction markers are MAM - IP3R and FACL4, MAM/ER – ATF6; and Mito – TOMM20 and HSPD1.

The cellular localization of PGRMC1 was probed using advanced subcellular fractionated of mouse hepatocytes from ex vivo livers to velocity separate mitochondrial and MAM fractions. Mouse livers were chosen over cultured cells for convenience of obtaining appreciable MAM yields. Livers isolated from 24-h fasted mice were used to isolate subcellular fractions enriched in MAMs, pure mitochondrial fractions, and ER-rich fractions. The fractions of interest were then probed with antibodies against known compartment-specific markers: mitochondria matrix – 60 kDa heat shock protein (HSPD1); mitochondria OM – mitochondrial import receptor subunit TOM20 homolog (TOMM20); and MAM-resident proteins - inositol 1,4,5-trisphosphate-gated calcium (IP3R) and long-chain-fatty-acid-CoA ligase 4 (FACL4); and ER/MAM-cyclic AMP-dependent transcription factor ATF-6 alpha (ATF6). Of note, we observed in our experiments that more ATF6, and less FACL4, was associated with MAMs when compared to the ER-enriched fraction (Fig. 3). Importantly, immunoblot analyses with PGRMC1-specific antibody revealed PGRMC1 in MAMs (lane 4) but not the pure mitochondrial or ER-enriched fractions (lanes 3 and 7), reflecting its predominant association with the MAMs. Interestingly, PGRMC1 signal was also observed in the nuclear and lysosomal/plasma membrane fractions (lanes 5 and 6). This could be due to lower purity of those specific fractions or altered localization to other cellular membranes as has been reported [16]. Overall, these results show that PGRMC1 is enriched at MAMs.

### 3.4. PGRMC1 metabolite interactions

**Fig. 4.**
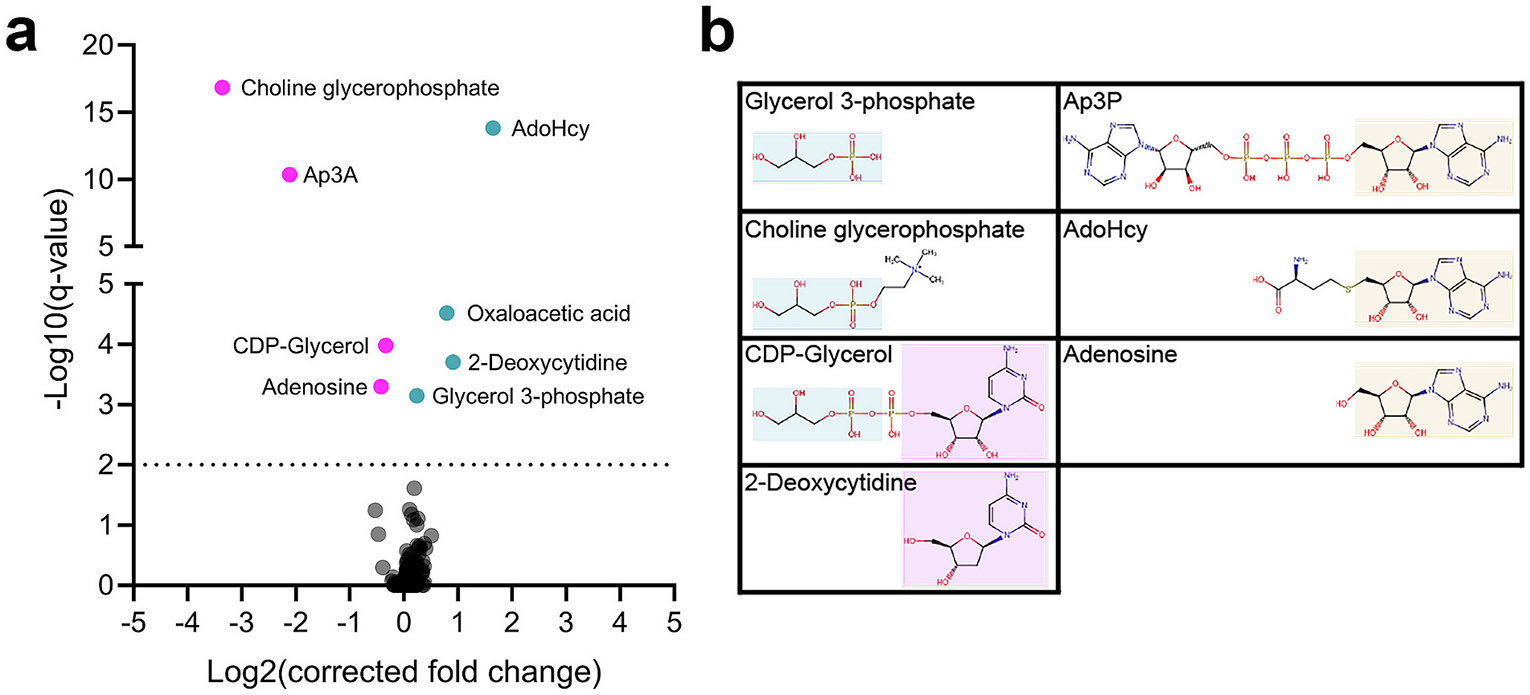
MIDAS analysis of PGRMC1. (a) Representative volcano plots of significant metabolite interactions of PGRMC1. Metabolites with a positive fold change are shown in teal and those with negative fold changes are shown in magenta. PGRMC1 protein was assayed by triplicate equilibrium dialysis and technical triplicate flow injection analysis-mass spectrometry injections. Significant PGRMC1 metabolite interactions identified by MIDAS are labeled and have a q < 0.01 (dotted line). (b) Metabolites identified by MIDAS analysis with common signatures highlighted in blue – glycerol 3-phosphate, pink – cytidine, and yellow - adenosine. Abbreviations: AdoHcy, S-(5-adenosyl)-L-homocysteine; Ap3A, diadenosine triphosphate; and CDP-glycerol, cytidine diphosphate-glycerol.

Mass spectrometry integrated with equilibrium dialysis for the discovery of allostery systematically (MIDAS) analysis was carried out to help identify metabolites which interact with PGRMC1 either as substrates or regulators. This screen used only soluble metabolites, hence heme, other metalloporphyrins, or other hydrophobic molecules are not included in the analysis. The MIDAS screen of PGRMC1 for metabolite interactions resulted in eight hits (Fig. 4a). Four of the metabolites showed a positive fold change (teal) indicating a possible direct and non-covalent interaction. Four of the metabolites had a negative fold change (magenta) which signifies a higher affinity interaction such as substrate to product conversion or covalent attachment of the metabolite to PGRMC1. There is a clear glycerol 3-phosphate signature as three of the significant hits share the common substructure (glycerol 3-phosphate, CDP-Glycerol, and choline glycerophosphate). A purine nucleoside/nucleotide signature is also apparent in several metabolites (S-(5-Adenosyl)-L-homocysteine (AdoHcy), adenosine, and diadenosine triphosphate (Ap3P)), as well as a pyrimidine signature in two metabolites (cytidine diphosphate-glycerol and 2-deoxycytidine) (Fig. 4b). Of interest, and related to MAMs, is the glycerol-3-phosphate moiety since the synthesis of two major phospholipids [59], phosphatidylethanolamine and phosphatidylcholine, is known to occur at MAMs [60]. Thus, these may be related to the cellular localization or regulation of PGRMC1.

### 3.5. Metabolomics analysis of PGRMC1 KO in an erythroid cell culture model

In a previous study, we showed that treatment of mammalian erythroid cell culture models with AG-205, a small molecule inhibitor of PGRMC1 that has been suggested to displace heme from PGRMC1 [61,62], resulted in decreased hemoglobinization in differentiated cells in a dose dependent manner [7]. This decrease in hemoglobin in differentiated cells could be partially rescued by overexpression of exogenous PGRMC1. While a phenotype was observed in differentiated cells, the exact nature of the interaction between AG-205 and PGRMC1 remains unclear and a direct effect of AG-205 on PGRMC1 function has not been demonstrated. Moreover, it is also unclear to what degree AG-205 influences PGRMC2 and what contribution this may have to the observed hemoglobinization phenotype. Additionally, recent work by several research groups has shown AG-205 to produce phenotypes non-specific to PGRMC1 [63-65]. Because of these factors, a different, more direct approach was sought to assess the function of PGRMC1 in erythroid cells. This was accomplished by the creation of a KO by CRISPR-Cas9 genome editing of PGRMC1 in a human erythroid cell line, K562 human myelogenous leukemia. This strategy allows for specific perturbation of PGRMC1 without the potential pleotropic effects of AG-205 treatment.

**Fig. 5.**
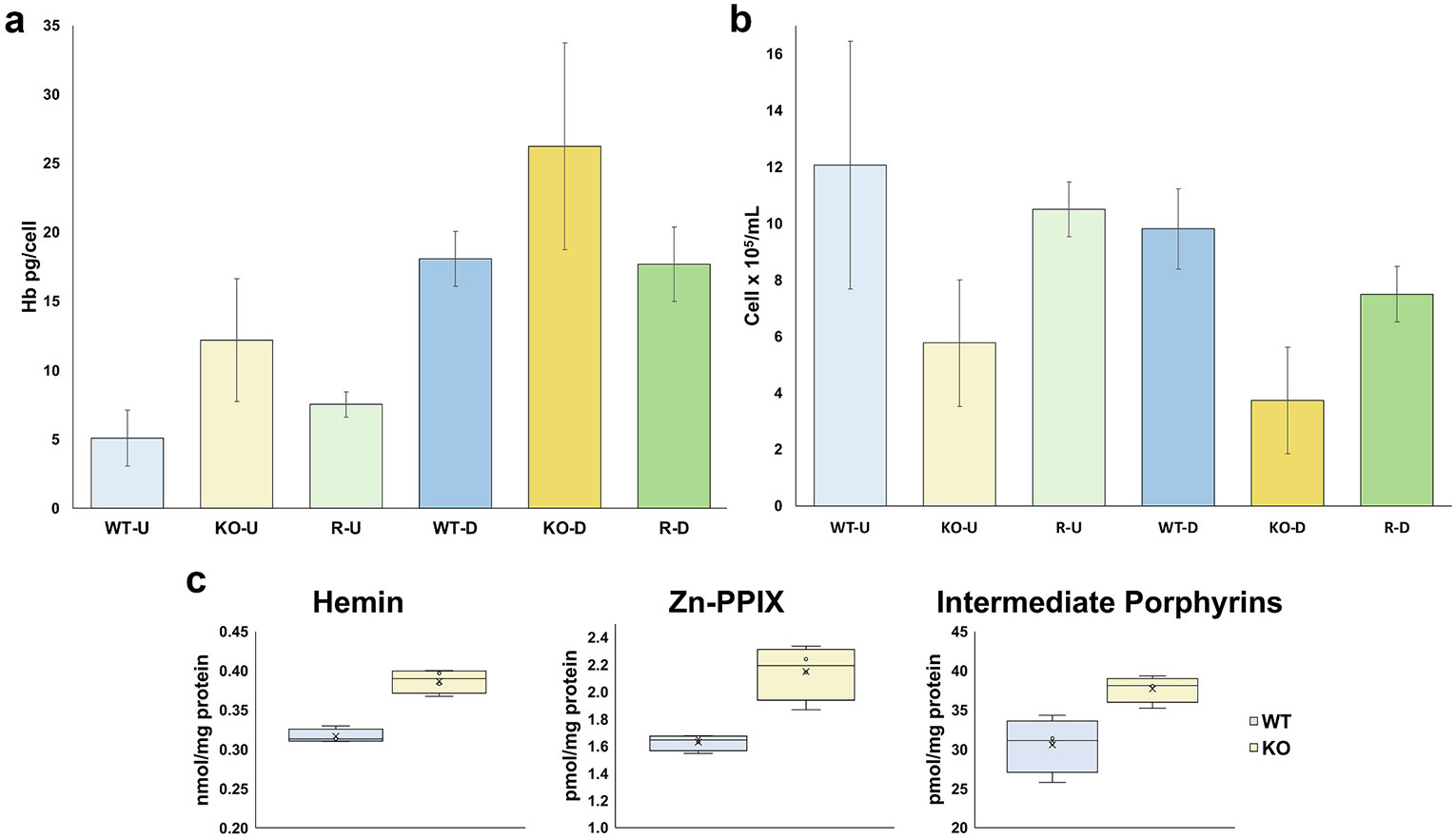
PGRMC1 KO increases heme synthesis and decreases cell count. (a) Hemoglobin (Hb pg/cell) levels in PGRMC1 KO (KO) cells are increased relative to wild-type (WT) in both undifferentiated (U) and differentiated (D) cells. Rescue (R) via PGRMC1 expression returns hemoglobin levels close to that of WT. *P* < 0.01 was found for WT-U vs KO-U and WT-D vs KO-D. *P* < 0.05 was found for WT-U vs R-U, KO-U vs R-U, and KO-D vs R-D. For WT-D vs R-D no statistically significant difference was found. (b) PGRMC1 KO (KO) decreases cell counts in both undifferentiated (U) and differentiated (D) cell culture. Rescue (R) with exogenous PGRMC1 expression partial corrects the cell count phenotype. *P* < 0.01 was found for WT-U vs KO-U, KO-U vs R-U, WT-D vs KO-D, WT-D vs R-D, and KO-D vs R-D. For WT-U vs R-U no statistically significant difference was found. (c) Hemin, Zn-PPIX and intermediate porphyrins were all increased in undifferentiated PGRMC1 KO K562 cells compared to wild-type (WT) cells. *P* < 0.01 was found for KO vs WT for hemin and Zn-PPIX and *P* < 0.05 for Intermediate Porphyrins.

The PGRMC1 KO out was performed using two target sites simultaneously (supplemental Fig. S1a) and consists of an approximately 110 bp deletion resulting in frame shift and ablation of protein expression (supplemental Fig. S1b,c) relative to total protein. To determine the effect that PGRMC1 ablation would have on hemoglobinization we measured hemoglobin in both undifferentiated and differentiating cells. PGRMC1 KO cells showed approximately 2.4-fold increased hemoglobin per cell relative to wild-type in undifferentiated cells and an approximately 1.4-fold increase in differentiated cells (Fig. 5a). In addition, PGRMC1 KO cells exhibited slower growth compared to wild-type cells (Fig. 5b). To confirm the specificity of these phenotypes, PGRMC1 KO cell lines were transfected with a vector expressing human PGRMC1. KO cell lines expressing PGRMC1 from the rescue vector showed hemoglobin levels returned to near those of wild-type cells (Fig. 5a). The growth phenotype was also partially rescued (Fig. 5b). The slightly slower rate of growth observed in the rescue cells relative to wild-type cells is likely attributable to the puromycin resistance cassette and antibiotic challenge present in the rescue condition but not in the wild-type cells. Hemoglobin in these cells was determined via spectroscopy through an increase in absorbance at 410–415 nm as previously described [46] with the assumption that the bulk of absorbance in this wavelength range corresponds to heme bound to hemoglobin. However, free heme, porphyrins, and metalated porphyrins also absorb in this range.

To further characterize the metabolites which could contribute to the 410–415 nm absorbance, hemin (ferric heme), protoporphyrin IX, Zinc-protoporphyrin IX (Zn-PPIX), and total porphyrins in undifferentiated wild-type and PGRMC1 KO K562 cells were determined and quantitated via ultra-performance liquid chromatography. We found hemin was increased 1.2-fold, Zn-PPIX 1.3-fold, and total porphyrins 1.2-fold in the PGRMC1 KO compared to wild-type cells (Fig. 5c). No appreciable amounts of PPIX were found in the cells in question. These finding suggest that overall heme biosynthesis is increased in PGRMC1 KO cells possibly through the dysregulation of FECH or altered heme trafficking due to the absence of PGRMC1.

**Fig. 6.**
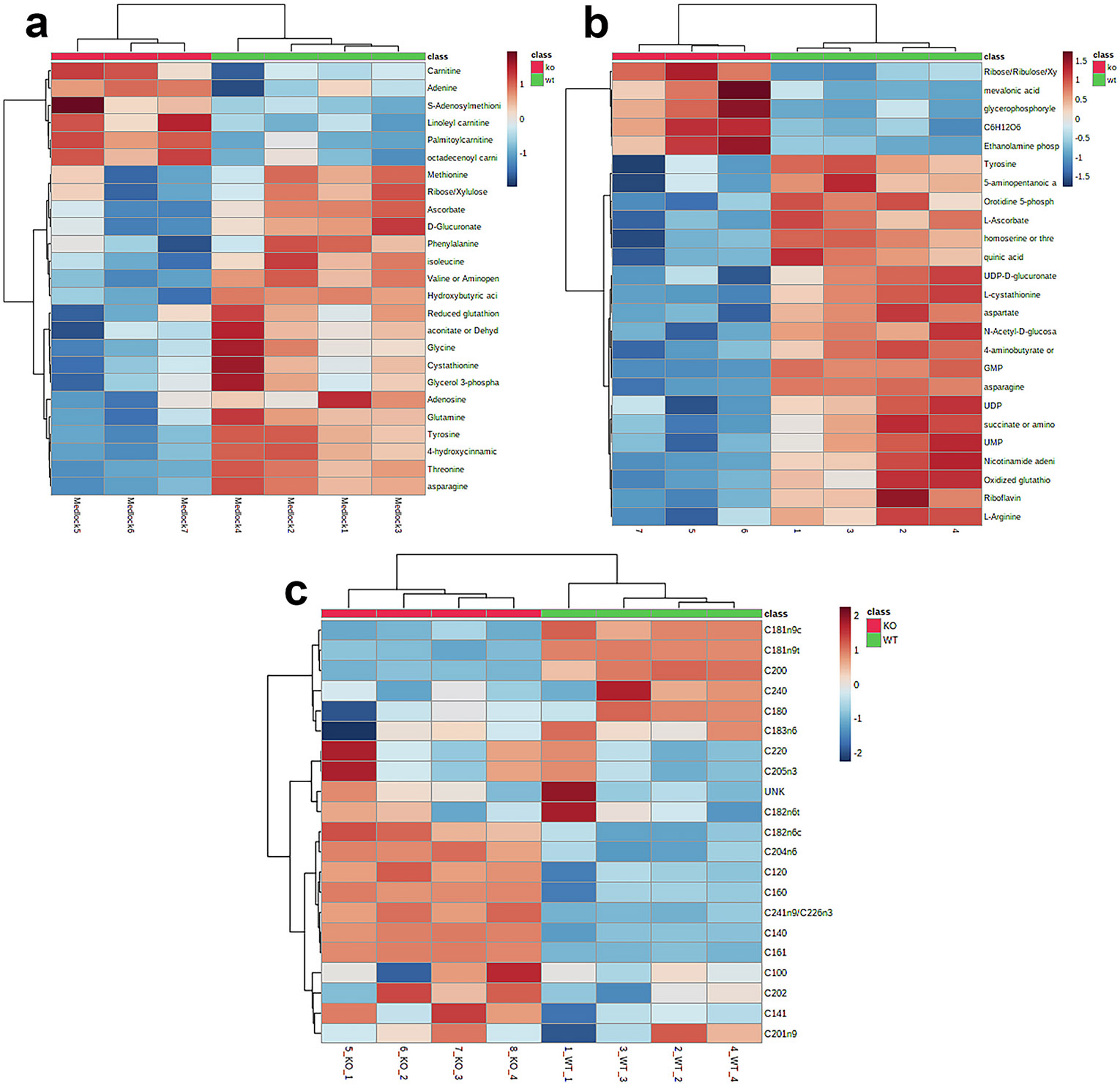
Metabolite alterations in PGRMC1 KO cells. (a) Heat maps of 25 most changed metabolites identified by LC-MS in positive-mode and (b) negative mode. (c) Heat map 25 most changed fatty acids identified by GC-FID. Analysis performed by MetaboAnalystR [48] on WT *n* = 4 and KO *n* = 3 for LC-MS and n = 4 for GC-FID.

To determine if other metabolites differences exist in the PGRMC1 KO cells, metabolomics analysis was carried out. Metabolites connected to heme metabolism in erythroid cells, specifically glutamine and glycine, were decreased in PGRMC1 KO cells as compared to wild-type (Fig. 6a). Both amino acids serve as precursors for heme [66] and, thus, as heme synthesis increases in the PGRMC1 KO cells overall cellular levels of these metabolites would decrease as observed. Several other amino acids correlated with hemoglobin levels, specifically isoleucine and valine [67], were also decreased in the PGRMC1 KO cells, likely due to increased hemoglobin production. Interestingly, several metabolites involved in cellular redox homeostasis are altered. Both oxidized and reduced glutathione levels are decreased and S-adenosylmethionine is increased (Fig. 6a and b). These changes are potentially a result of increased oxidative stress in the cell due to increased heme and heme precursor levels [68]. These findings together support the metabolic consequences of increased heme synthesis in PGRMC1 KO cells.

Other noted changes in metabolite levels in the PGRMC1 KO cell are related to lipid and phospholipid metabolism. It is established that synthesis of the most abundant phospholipids, specifically phosphatidylcholine and phosphatidylethanolamine [59], occurs at MAMs and require transfer between the mitochondria and ER [22]. For phosphatidylethanolamine, two pathways for its synthesis exist in mammalian cells, the CDP-ethanolamine pathway which occurs in the cytosol and ER and the decarboxylation of phosphatidylserine which occurs in the mitochondria. Both pathways require MAMs in different ways for the movement of phospholipids between compartments [60]. In PGRMC1 KO cells, both ethanolamine phosphate and glycerophosphorylethanolamine are increased (Fig. 6b) indicative of abnormalities in phospholipid metabolism. Ethanolamine phosphate is a substrate for the enzyme catalyzing the rate-limiting step of the CDP-ethanolamine pathway [69] and, thus, would be expected to accumulate if further metabolism of phosphatidylethanolamine at MAMs is affected.

Long chain acyl-carnitines (Linoleyl-, palmitoyl- and octadecenoyl-) as well as carnitine, are elevated in PGRMC1 KO cells (Fig. 6a). Also increased in the KO cells are saturated medium chain length fatty acids, including C12:0, C14:0, and C16:0, while most of the saturated long chain fatty acids are decreased (C18:0, C20:0, and C24:0) (Fig. 6c). No clear trends were observed for unsaturated and polyunsaturated fatty acids. The current data are insufficient to fully explain the metabolic basis for the observed changes but possibilities include decreased mitochondrial beta oxidation, increased mitochondrial fatty acid synthesis [70], decreased ER and mitochondrial utilization for phospholipid synthesis, or decreased ER use for cholesterol esterification. PGRMC1 has been long connected with cholesterol synthesis via its interaction with cytochrome P450 51A1 [11,49]. A clue from the current data suggesting that cholesterol metabolism may play a role is the finding that mevalonic acid, an intermediate in cholesterol metabolism, is increased in the PGRMC1 KO cells. A decrease in cholesterol production and esterification would result in some of the alterations in lipid levels, but are not sufficient to explain all the changes described herein.

Another process which occurs at MAMs is the synthesis of coenzyme Q [71]. This process requires tyrosine to produce the quinone head group and mevalonic acid for the hydrophobic tail [72]. Previous work investigating the proteins of the coenzyme Q metabolon, specifically enzymes which modify the quinone head group, demonstrated that defectives in ER-mitochondria contact sites resulted in accumulated intermediates of the pathway. While the precise role of the MAMs in coenzyme Q synthesis is unclear, they may exist to coordinate synthesis and convergence of the hydrophobic tail (derived from mevalonic acid) and the quinone head group (derived from tyrosine). In the PGRMC1 KO cells, both precursors of coenzyme Q are altered. As mentioned above, mevalonic acid levels are increased (Fig. 6b), while tyrosine levels are decreased (Fig. 6a). Although the observed changes in each of these metabolites are opposite, the dysregulation of these separate processes that converge at the coenzyme Q metabolon may explain the change in PGRMC1 KO cells.

Overall, the metabolomic analysis of PGRMC1 KO cells is consistent with roles of PGRMC1 in heme homeostasis as well as phospholipid and lipid metabolism, with possible connections to coenzyme Q metabolism which warrant further investigation. Both the phospholipid and coenzyme Q pathways provide an additional link of PGRMC1 to MAMs. In addition, several other metabolites (Fig. 6a and b), including carbohydrates and amino acids, are altered in PGRMC1 KO cells, though their link to heme or MAMs is unclear at present.

## 4. Conclusions

Herein we present data further linking PGRMC1 to both MAMs and heme trafficking as well as data supporting the role of MAMs in heme trafficking to the nucleus. Subcellular fractionation of MAMs from mouse liver clearly shows that PGRMC1 is enriched at MAMs as suggested by multiple proximity labeling studies [18-20]. In addition, analysis of the PGRMC1 interactome revealed that 25 % of the protein partners are either putative or validated MAM proteins with enrichment of biological process which occur at MAMs. Many of these processes, including lipid metabolism and calcium homeostasis, have been connected to PGRMC1 though details of PGRMC1’s role are sparse [9]. The localization and importance of PGRMC1 at MAMs is further underscored via metabolomic analysis of PGRMC1 KO cells which demonstrated altered levels of metabolites related to metabolic pathways which occur at MAMs including phospholipid, cholesterol, and coenzyme Q metabolism [23,71]. The connection of heme trafficking by PGRMC1 to these other metabolic pathways is unclear, but linked via the localization of PGRMC1. Further work is necessary to determine the connection between heme and other MAMs pathways.

In addition to the localization of PGRMC1 at MAMs, our data have further defined the role of PGRMC1 in heme homeostasis and trafficking. Previously PGRMC1 was identified as a component of the mitochondrial heme metabolon [7,31], which is found at mitochondrial inner and outer membrane junctions [68]. The mitochondrial heme metabolon is necessary for regulating heme synthesis as increased levels of its core proteins result in increased levels of porphyrin precursors and mis-metalated porphyrins [31]. These alterations are reminiscent of those seen in the pathological condition X-linked protoporphyria [73] and suggest that PGRMC1 should be examined as a possible modifier protein in some porphyrias. Findings presented herein support a prominent role for PGRMC1 as a core component of the mitochondrial heme metabolon as ablation of PGRMC1 results in increased levels of heme, porphyrins, and Zn-PPIX, similar to those observed for metabolon disruption. Likewise, alterations in levels of metabolites required for in heme synthesis, specifically glutamine and glycine, are observed in PGRMC1 KO cells supporting the role of PGRMC1 in regulating heme synthesis. These findings, along with those obtained from studies in the yeast model showing altered heme trafficking in *dap1*Δ cells, reinforce our previous findings that PGRMC1 functions in cellular heme homeostasis. Further analysis of the FECH and PGRMC1 core interactomes shows several proteins in common that have been identified at MAMs, specifically ABCD3, ATAD1, PGRMC2, and RHOT1. The submitochondrial localization of the mitochondrial heme metabolon at inner and outer membrane junctions with MAMs is reminiscent of the coenzyme Q metabolon [71]. Thus, the findings presented above support a role for PGRMC1 at MAMs interacting with the mitochondrial heme metabolon regulating synthesis and trafficking of heme from mitochondria to the nucleus via the ER as supported by studies in yeast [26] and PGRMC2 in adipose tissue [5]. Further studies focused on understanding the structure of the mitochondrial heme metabolon as well as work to investigate the consequences of stabilization or disruption of MAMs on heme synthesis are needed to fully dissect and understand the mechanisms of heme trafficking.

## Supplementary Material

Supplemental Figures 1 and 2

Table S1: CRAPome Filtered Data

Table S1: Affinity Purification Raw Data

Table S3: Biological Process Enrichment

Table S4: Abbreviation Table

Supplementary data to this article can be found online at https://doi.org/10.1016/j.jinorgbio.2025.113093.

## Data Availability

The protein interactions from this publication have been submitted to the IMEX (https://www.imexconsortium.org/) consortium through IntACT [74] and are assigned the identifier IM-30504. Metabolomics data obtained in this study will be accessible at the NIH Common Fund‘s NMDR (supported by NIH grant, U01-DK097430) website, the Metabolomics Workbench, https://www.metabolomicsworkbench.org.
