# Supplementary material for: Progesterone receptor membrane component 1 (PGRMC1) regulates Heme trafficking through mitochondria-ER junctions
Source: J Inorg Biochem. Author manuscript; Available in PMC 2026 Mar 10. (PMC12973532; doi:10.1016/j.jinorgbio.2025.113093)
Supplement: Supplemental Figures 1 and 2 [file NIHMS2137147-supplement-Supplemental_Figures_1_and_2.docx]

**Supplemental Figures S1 and S2 for**

**PGRMC1 Regulates Heme Trafficking through Mitochondria-ER Junctions**

Robert B. Piel, III^a,#,$^, Chibuike D. Obi^a,#^, Martonio Ponte Viana^b,§^, Mathilda M. Willoughby^c,&^, Osiris Martinez-Guzman^c,%^, Aaliyah Wadley^c,^^, Yasaman Jami-Alahmadi^d^, James A. Wohlschlegel^d^, Kevin Hicks^e,f^, Jared Rutter^e^, J. Alan Maschek^f,g^, J. Leon Catrow^g^, James Cox^e,g^, Amit R. Reddi^c,h^, Oleh Khalimonchuk^b,i,j^, Amy E. Medlock^a,k^*


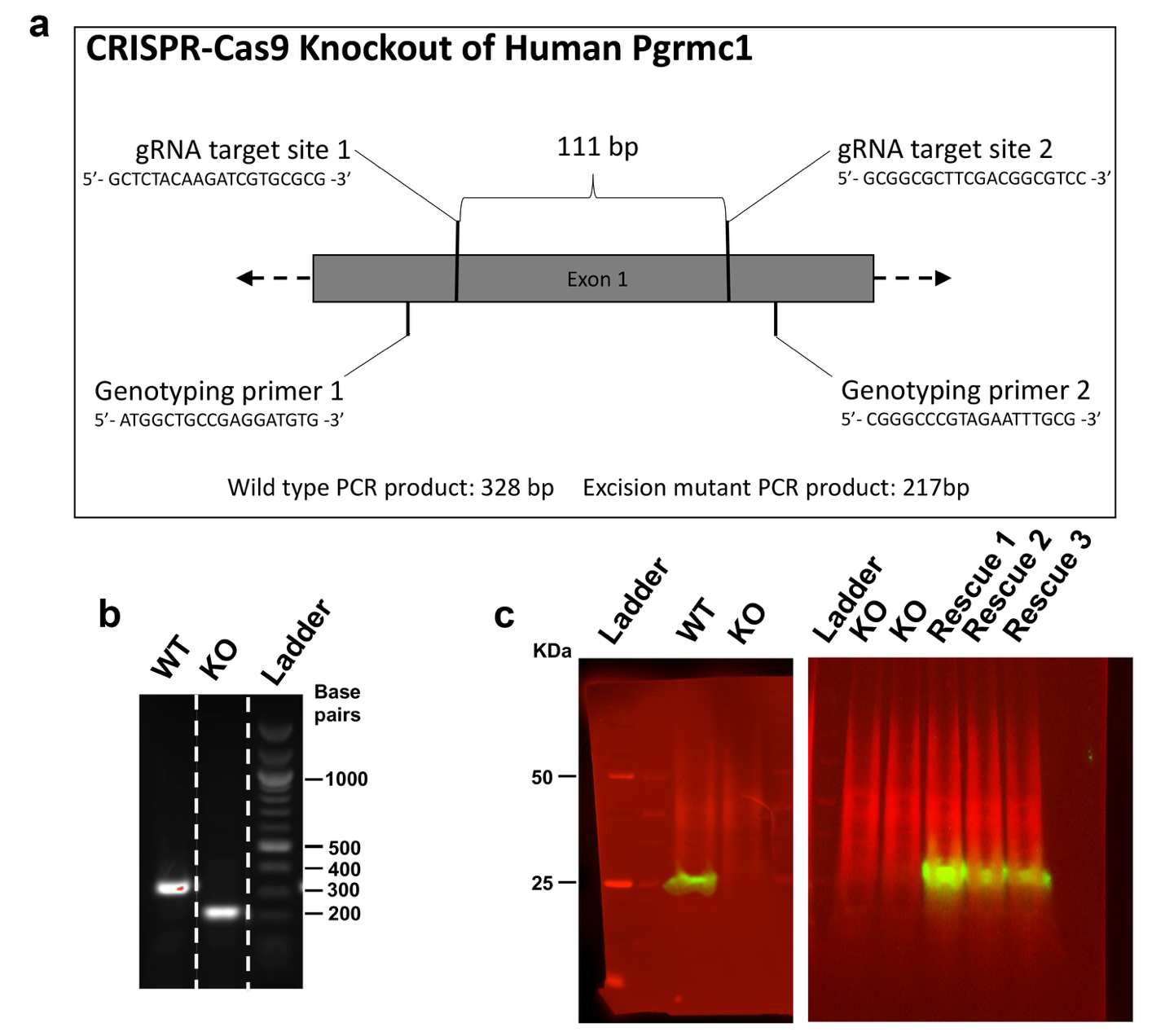


**Supplemental Figure 1. KO of Pgrmc1 in K562 cells.** (a) Diagram of CRISPR-Cas9 gRNA target sequences and genotyping strategy for PGRMC1. (b) Agarose gel showing PCR based genotyping of wild type (WT) and PGRMC1 KO K562 cells with 100 bp DNA ladder (c) Western blot of WT and KO cells showing ablation and rescue of PPGRMC1 expression (green) normalized to total protein (red) with ladder.


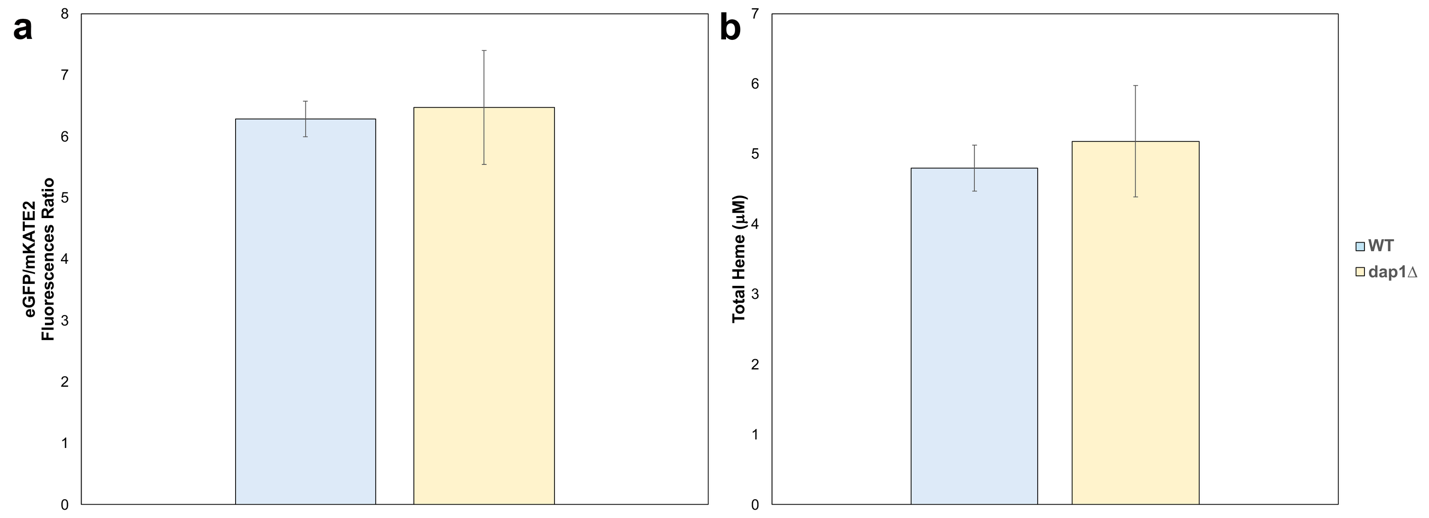


**Supplemental Figure S2. Steady state levels and total heme in *dap1*Δ cells.** (a) Labile heme levels in the cytosol were measured using the HS1 heme sensor. (b) Total cellular heme levels were measured using the oxalic acid method.
